# Identifying the factors that influence surgeon's compliance with excisional margins of non-melanoma skin cancer

**DOI:** 10.1371/journal.pone.0204330

**Published:** 2018-09-27

**Authors:** Jennifer Marchetti Cautela, Alice Mannocci, Camilla Reggiani, Flavia Persechino, Federica Ferrari, Elena Rossi, Erika Passini, Pierantonio Bellini, Marco Meleti, Sara Wertzberger Rowan, Cristina Magnoni

**Affiliations:** 1 Department of Dermatology, Head and Neck Skin Cancer Service, Modena and Reggio Emilia University, Modena, Italy; 2 Department of Public Health and Infectious Diseases, “Sapienza” University, Rome, Italy; 3 Department of Head and Neck Surgery, Unit of Maxillofacial Surgery, Modena and Reggio Emilia University, Modena, Italy; 4 Department of Medicine and Surgery, Unit of Dentistry, University of Parma, Parma, Italy; 5 Independent Researcher, Florence, Italy; University of Queensland Diamantina Institute, AUSTRALIA

## Abstract

The rising incidence of Non Melanoma Skin Cancers (NMSC) leads to a high number of surgical procedures worldwide. The strict compliance with international guidelines with regard to excisional margins may help decrease the number of re-excision procedures and reduce the risk of NMSC recurrence. The aim of this study was to investigate the prevalence of excisional margins as recommended by the European Academy of Dermatology and Venereology (EADV) and the European Dermatology Forum (EDF) guidelines, and the factors (demographic or clinical) that influence surgeons’ compliance with these guidelines.This was a prevalence study looking at surgical excisions of NMSCs performed over a period of 2 years (2011–2012). A sample size of 1669 patients was considered. Definition of excisional margins recommended by the international guidelines (EADV and EDF) were used as point of reference for the analysis. Tumor and histologic specimen size were calculated ex vivo by 5 different pathologists. The size of skin specimens was measured with a major axis and a minor axis. The same was done for the tumor present on the skin specimens. The differences between the major and minor axes of surgical specimen and tumor were calculated. These differences were subsequently divided by two, hypothesizing that the lesion had the same distance from the margins of the surgical specimen.

The differences obtained were named “Delta”, the formulas applied being the following:Delta major = (major axis specimen—major axis tumor)/2; Delta minor = (minor axis specimen—minor axis tumor)/2.Results show a significant statistical difference, associated with factors such as: age of the patient, anatomical localization of the tumor, histological diagnosis, and surgeons’ experience.The identification of these factors sheds light on clinicians’ practice and decision-making regarding excisional margins. Hopefully a higher level of adherence to the guidelines can be achieved in the future.

## Introduction

Non-melanoma skin cancer (NMSC) is the most frequent malignant neoplasm in white populations and accounts for at least 80% of all skin cancer. NMSC consists mainly of basal-cell carcinomas BCC (70%) and squamous cell carcinoma SCC (20%) [1]. A continuous long-term increase of NMSC incidence has been reported worldwide by Eisemann et al [2], to the point that the phenomenon has been described with the expression “skin cancer epidemic” [3].

Although the mortality rate from NMSC is low, as reported by Leiter et al [1], such skin cancer impacts patients’ quality of life causing significant morbidity.

Surgical excision with pre-operatively identified margins is one of the most common and effective treatment strategies for basal-cell carcinomas (BCC) as well as for most squamous cell carcinomas (SCC).

The recommended excisional margins both for SCCs and BCCs are indicated by guidelines issued by specific Scientific Societies and Boards of Experts and they have substantially evolved in recent years. [4,5]

Insufficient compliance with recommended excisional margins among surgeons represents a very high risk of NMSC recurrence and re-excision for patient.

It is important to note that the presence (or absence) of tumor cells in the margins is among the prognostic features for recurrences of SCCs. When initial removal is incomplete, SCC is more likely to recur mostly locally or less frequently in regional lymph nodes. Approximately 75% of recurrences occur within two years and 95% within five years after initial diagnosis. [6]

Tumor recurrence is associated with incomplete excision also in the case of BCCs, even though it is not as high as might be expected. It ranges from 26 to 41% after 2 to 5 years of follow-up, and the maximum number of tumor recurrences has been detected in morphoeic and facial tumors. [7,8]

Moreover, when margins are involved, the recommendation is usually for re-excision or for a close clinical follow-up. This not only produces negative effects on patients but also causes an increase in additional healthcare costs.

In this context, the optimization of the surgical management of NMSC is of great importance in order to ensure the highest quality of surgical treatments and a subsequent optimal outcome for all patients.

Few data are available regarding surgeons’ compliance with recommended surgical treatment of NMSC.

The aim of the present retrospective evaluation is to identify factors, both demographic and clinical, that can affect the compliance with international guidelines (AEMR) regarding excisional margins in surgical treatment of NMSCs.

## Materials and methods

### Study design

This is an epidemiological analysis investigating (1) the prevalence of the size of excisional margins as recommended by the European Academy of Dermatology and Venereology (EADV) and the European Dermatology Forum (EDF) guidelines, and (2) the factors that influence surgeons’ compliance with these guidelines.

The duration of the study was a 2-year period (between January 2011 and December 2012). It took place at the Department of Dermatologic Surgery of the University Hospital of Modena, Italy.

The ethical committee (Comitato Etico dell’Area Vasta Emilia Nord, Policlinico di Modena, Via Largo del Pozzo 71, 41124) approved the study protocol and the revision of medical records (Prot. N. 99/17). There was no requirement on part of the ethical committee to obtain an informed consent to access retrospectively to the data.

The STrengthening the Reporting of OBservational Studies in Epidemiology (STROBE) was applied to perform the research [9]. STROBE consists of a checklist of 22 items that provide guidance on the reporting of observational studies, created by an international, collaborative initiative of epidemiologists, methodologists, statisticians, researchers and journal editors in order to facilitate critical appraisal and interpretation of results.

As a reference, the study used the guidelines issued in 2011 by the EADV for SCC management and the guidelines issued in 2006 by EDF for BCC management. According to such guidelines, a margin of 4–6 mm was suggested for low-risk SCCs, whereas an extended margin (≥6 mm, or even 10 mm or more) was recommended for high-risk tumors, particularly for those with risk of subclinical extension. With regard to BCCs, a 4–5 mm surgical margin was recommended for small (< 20 mm) well defined BCCs, while a wider margin (13–15 mm) was suggested for morphoeic and larger BCCs [10,11].

The surgical procedures analyzed in the study were carried out by 23 surgeons familiar with EADV and EDF guidelines.

### Inclusion and exclusion criteria

The number of surgical procedures analyzed was 1669. All patients with histological confirmed diagnosis of NMSCs were included in the study and all medical records of patients were retrieved for data analysis. Medical records reporting punch biopsies and histological diagnosis of melanomas were excluded from the study.

All the surgical excisions considered in the study were performed as standard surgical excisions followed by post-operative pathologic assessment of margins (conventional histology with paraffin-embedded definitive evaluation). All patients with hystological confirmed incomplete excision underwent re-excision according to the clinical procedure of the Department.

### Data collection

Information from medical records was extracted by six independent researchers. The medical records were accessed by study investigators in order to create a database for the study (S1 File). It was anonymized by appointing a number to each patient. The data extracted regarding tumors included histological diagnosis (BCC, SCC) and anatomical localization of the lesions, classified according to the guidelines previously reported. Localization of tumors was assessed according to 3 areas:

1. High-risk area (H): eyelids, eyebrows, periorbital, nose, lips, chin, mandible, pre-auricular and post-auricular skin, temples, ears, genitalia, hands, and feet.
2. Intermediate-risk area (I): cheeks, forehead, scalp, neck, and pretibial.
3. Low-risk area (B): trunk, and extremities.

Further data was retrieved regarding surgical reconstruction techniques—i.e. primary closure; grafting technique; advanced, rotational and transpositional flaps (respectively flaps A, R and T)—together with histological data on the presence of neoplastic cells on the surgical margins (no, yes deep, yes lateral, and yes both).

Patients were divided into three groups according to age, in order to reflect different clinical needs as follows: ≤75 years old, between 76 and 85 years old and ≥ 86 years old.

Surgeons were divided based on years of experience (<10; ≥10 years).

### Definition of excisional margins recommended by the international guidelines (EADV and EDF)

The international guidelines were used as the standard and as point of reference for the analysis of this study. The guidelines divide BCCs and SCCs in low risk (R-) and high risk (R+), depending on diameter and location of the tumor.

BCCs are considered at low or high risk as follows:

- R-: diameter <20 mm independently of the location.
- R+: diameter ≥20 mm independently of the location.

SCCs are considered at low or high risk as follows:

- R-: diameter <10 mm in area H.
- R-: diameter <20 mm in areas I and B.
- R+: diameter ≥10 mm in area H.
- R+: diameter ≥20 mm in areas I and B.

The risk criteria determine the excisional margins recommended by the guidelines as follows:

- = 5 mm in the case of BCCs R-.
- > 5 mm in the case of BCCs R+.
- 4–6 mm in the case of SCCs R-.
- 6–10 mm in the case of SCCs R+.

### Methods of analysis of excisional margins

Tumor and histologic specimen sizes were evaluated ex vivo by 5 different pathologists. Skin specimen has been described as a round or ellipsoidal area containing the tumor [12]. The size of skin specimens was measured with a major axis and a minor axis. The same was done for the tumor present on the skin specimens.

In order to measure the excisional margins we calculated the difference between the major and minor axes of the surgical specimen and those of the tumor. The differences between the major and minor axes of surgical specimen and tumor were calculated. These differences were subsequently divided by two, hypothesizing that the lesion had the same distance from the margins of the surgical specimen. Major and minor axes were measured in millimeters (mm).

**Fig 1:**
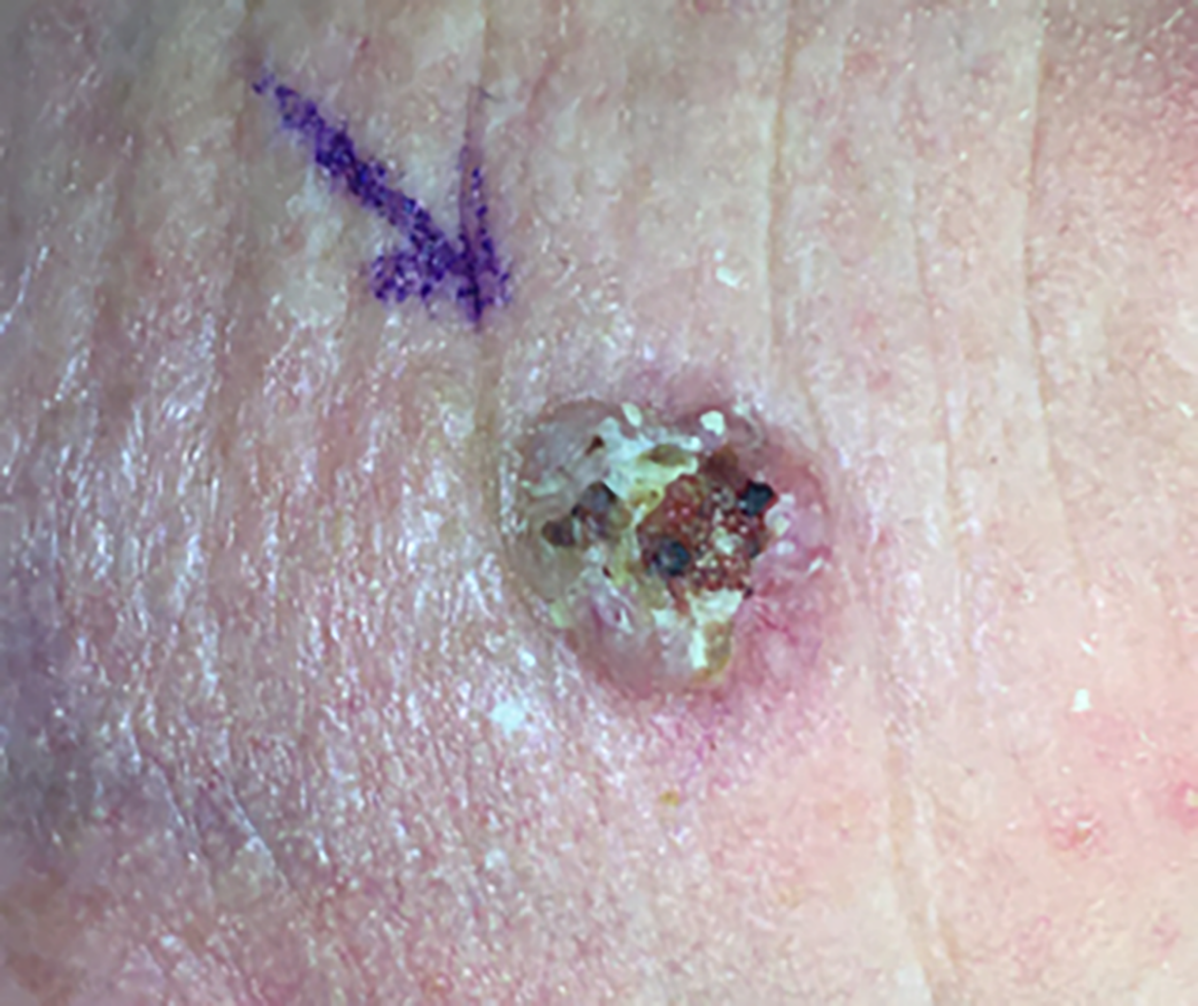
Squamocellular carcinoma of the forehead. Preoperative view.

**Fig 2:**
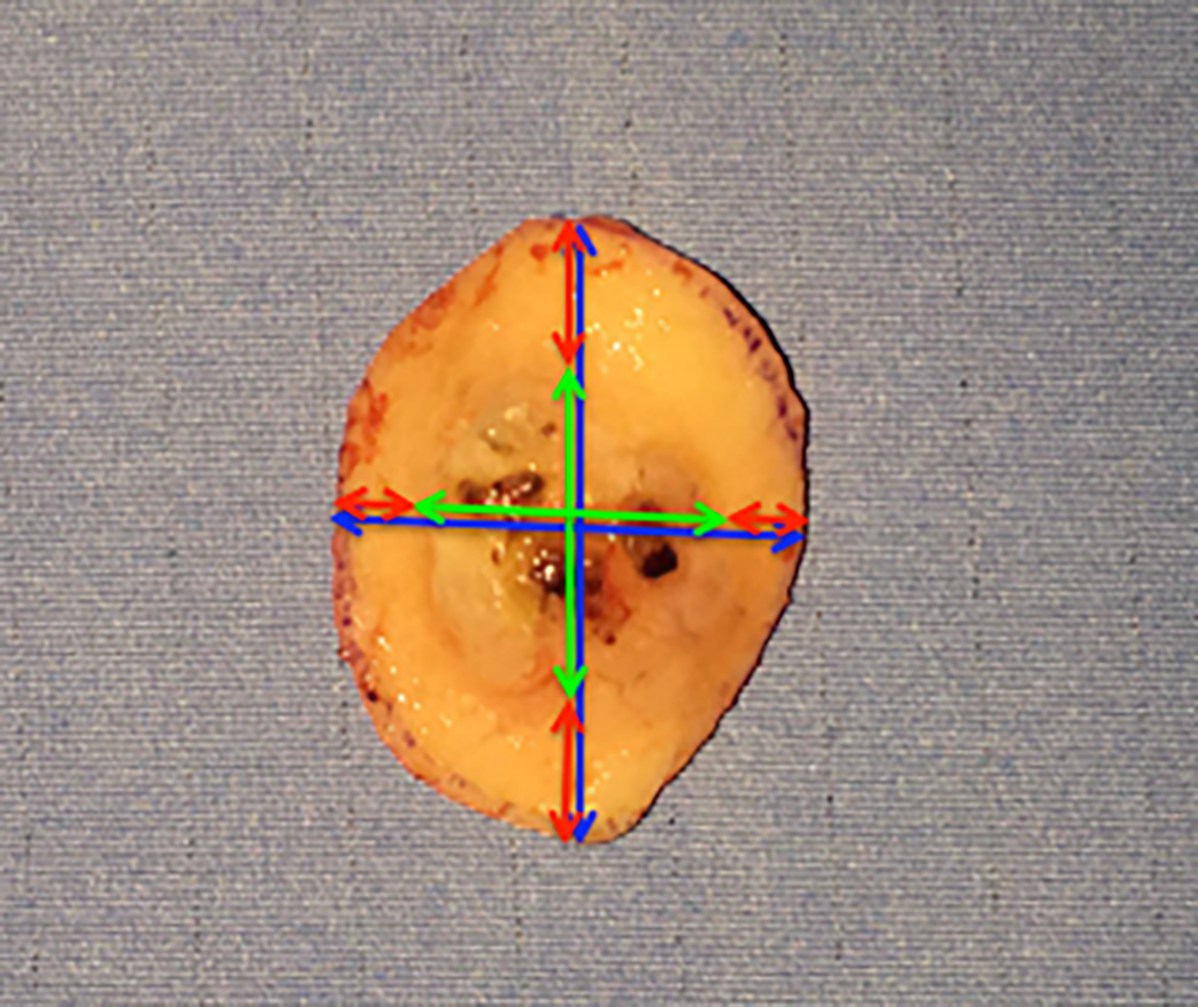
Squamocellular carcinoma of the forehead. Postoperative view of the skin specimen. The size of skin specimens was measured with a major axis and a minor axis (blue arrows). The same was done for the tumor present on the skin specimens (green arrows). The differences between the major and minor axes of surgical specimen and tumor was calculated to obtain the Deltas (red Arrows).

The differences obtained were named “Delta” (Figs 1 and 2), the formulas applied were the following:

$$Delta\;major=\frac{{axis\;major}_{speciment}-{axis\;major}_{tumor}}{2}$$

$$Delta\;minor=\frac{{axis\;minor}_{speciment}-{axis\;minor}_{tumor}}{2}$$

In order to take into account the presence of the shrinkage phenomenon, an enlargement correction analysis was used.

The shrinkage phenomenon is widely recognized in dermatopathology as the shrinking of the soft tissue when excised. A mean shrinkage of 17% in the length and 9.5% in the width had been considered on the histologic specimen dimension according to Blasco-Morente et al [13].

The presence of neoplastic cells on the surgical margins (no, yes deep, yes lateral, and yes both) was referred to as incomplete excision (Figs 1 and 2).

### Statistics

Qualitative variables have been presented as frequency distribution and percentage, whereas the quantitative ones have been presented as mean, SD, and relative Confidence Interval at 95% (95% CI).

Correlation coefficient had been applied to assess the relationship between the two Deltas.

Univariate analysis has been used to examine the significant difference of the mean value, SD, and CI 95% of both Delta major and Delta minor in different clinical and demographic categories. A similar analysis was carried out to evaluate the difference between these categories and the adherence to excisional margins recommended by the international guidelines (AEMR) looking at both Delta major and Delta minor. Two logistic regression models have been constructed using the results of univariate analysis. A regression model determines whether the proposed relationship between the response variable (in this study “AEMR referred to Delta minor” and “incomplete excision”) and a set of predictors is statistically reliable. The independent variables (predictors) in this study are demographic and clinical variables and they have been chosen on the basis of the p-value obtained in the univariate analysis (p<0.2).

A conditional forward stepwise was applied to select the significant variables. A Hosmer Lemeshow’s test was computed to evaluate the goodness of fit of the models.

All analyses were conducted on SPSS 19.0 (SPSS Inc., Chicago, IL USA), adopting p <0.05 for two-tailed tests.

## Results

### Description of the sample

One thousand six-hundred and sixty-nine procedures were included in the present evaluation. 37.8% of specimens analyzed belonged to female patients (N = 593), the mean age being 73.5 years (SD = 13.5, min = 23, and max = 102).

One thousand two-hundred and eight (72%) procedures were BCCs, while the remaining 461 cases (28%) were SCCs.

The majority of reconstructive procedures were primary closure (N = 1127, 67.5%) followed by grafts (N = 207, 12.4%), advancement flaps (flaps A) (N = 143, 9%), rotation flaps (flaps R) (N = 140, 8%), and transposition flaps (flaps T) (N = 52, 3%).

Tumors were mainly located in the B area (N = 611, 37%) followed by H (N = 533, 32%), and I (N = 525, 32%).

The percentage of high-risk (R+) intervention was 26% (N = 435).

The geometrical shape of surgical specimens was elliptical in 97% of cases (N = 1612) and round in the remaining cases (3%, N = 57).

Surgical specimens major axis had a mean size of 32.7 mm, SD = 16.5 (N = 1668). Minor axis had a mean size of 18.6 mm, SD = 11.4 (N = 1669).

The major axis of the tumors had a mean size of 13.1 mm, SD = 0.96 (cases valid N = 1662). The minor axis had a mean size of 10.9 mm, SD = 0.8 (N = 1590).

The differences between the major and minor axes of surgical specimen and that of the tumor are called respectively Delta major and Delta minor. Delta major was 11.8 mm (SD = 7.3mm with N = 1660 number of total lesions considered and CI95%: 1.2–1.2) and Delta minor was 4.3 mm (SD = 3.9 with N = 1587, CI95%: 4.2–4.5). Difference between Deltas was statistically significant (p<0.05). The Pearson’s correlation coefficient between Deltas was r = 0.462 (p<0.001).

### Factors influencing excisional margins—Univariate analysis

Univariate analyses describe the association between the mean value, SD, and CI95% of both excisional margins (Delta major and Delta minor) and clinical and demographic variables (see Table 1). Both Deltas were associated to patients’ characteristics: mean values were significantly lower in female and younger patients. The H area tumors had a lower mean value for both Deltas, compared to tumors of the B and I area (p<0.001). Primary closure showed the higher mean Delta major in comparison with the other ones (p<0.001); on the contrary, the graft technique had the higher mean Delta minor (p<0.001). It is worth reporting that the averages of Delta minor were highest in SCCs and in surgeons having more than 10 years of experience (p<0.001).

**Table 1:** Univariate analysis for Delta mean (major and minor) versus clinical variables and demographic characteristics.

Variables		Delta major(mm)^a^						Delta minor(mm) ^a^					
		N	mean	SD	CI95% (mean)		p^b^	N	mean	SD	CI95% (mean)		p^b^
					Low	Upp					Low	Upp	
Patient gender	M	990	12.6	7.7	12.2	13.1	**<0.001**^**c**^	990	4.5	4.0	4.3	4.8	**0.019**^**d**^
	F	593	10.5	6.3	10.0	11.0		593	4.1	3.6	3.8	4.3	
Patient age	≤75	763	11.3	6.8	10.9	11.8	**0.018** ^e^	763	3.7	3.2	3.5	3.9	**<0.001** ^**c**^
	76–85	515	12.2	7.4	11.6	12.9		515	4.7	3.8	4.4	5.0	
	≥86	305	12.4	7.9	11.5	13.3		305	5.4	5.2	4.8	5.9	
Anatomical localization of the lesions	I	522	10.5	6.1	10.0	11.0	**<0.001**^e^	502	4.5	3.7	4.2	4.8	**<0.001** ^e^
	B	610	11.4	6.1	10.9	11.9		574	3.8	3.7	3.5	4.1	
	H	528	7.2	4.9	6.7	7.6		511	3.4	2.7	3.2	3.6	
Diagnosis	BCC	1202	11.7	7.0	11.3	12.0	0.126 ^**c**^	1152	4.1	3.4	3.9	4.3	**<0.001** ^**c**^
	SCC	458	12.3	7.9	11.6	13.0		435	5.0	4.7	4.6	5.4	
BCC risk	R+	183	14.7	8.7	13.4	16.0	**<0.001**^**f**^	157	4.9	4.3	4.3	5.6	**<0.001**^**f**^
	R-	1477	11.4	7.0	11.1	11.8		1427	4.3	3.8	4.1	4.5	
SCC risk	R+	183	12.1	8.3	10.8	13.3	0.979^**f**^	174	6.6	5.9	5.7	7.5	**<0.001**^**f**^
	R-	1477	11.7	7.1	11.4	12.1		1412	4.1	3.5	3.9	4.3	
Surgical reconstruction techniques	flap A	143	6.3	5.1	5.5	7.2	**<0.001**^e^	139	3.4	2.3	3.1	3.8	**<0.001** ^e^
	flap R	139	6.1	4.1	5.5	6.8		135	3.7	2.1	3.3	4.1	
	flap T	51	7.1	3.7	6.0	8.1		48	4.5	3.5	3.4	5.5	
	graft	207	8.9	6.2	8.1	9.7		200	6.1	5.3	5.4	6.9	
	primary closure	1120	11.0	5.9	10.1	11.3		1065	3.5	3.2	3.3	3.7	
Surgeon experience (years)	<10	663	11.9	6.9	11.4	12.5	0.451 ^**d**^	663	3.8	3.1	3.6	4.0	**<0.001** ^**c**^
	≥10	920	11.8	7.5	11.3	12.3		920	4.7	4.3	4.4	5.0	

^a^ Delta corrected: it was computed considering the half difference between the axis of the surgical specimen and the lesion with the shrinkage corrections (see methods).

^**b**^ p-value of tests hypothesis: The null hypothesis was that the groups considered in the “Variables” column have the same mean.

^c^ p-value of t-student test with equal variances not assumed.

^d^ p-value of t-student test with equal variances assumed.

^e^ p-value of ANOVA test.

^f^ p-value Mann-Whitney test.

The averages of Delta minor were highest in surgeons having more than 10 years of experience (p<0.001) and with the histological diagnosis of SCCs

It is worth reporting that a significant difference was found between the BCC high risk group (R+) versus low risk (R-) for delta minor: the mean BCC high risk = 4.9mm (SD = 4.3) and the mean of BCC low risk = 4.3mm (SD = 3.8) with a p<0.001.

Concerning the SCC, the groups R+ versus R- shown a significant difference for delta minor (p<0.001): the mean SCC high risk = 6.6mm (SD = 5.9) and the mean of SCC low risk = 4.1mm (SD = 3.5) with a p<0.001.

### Factors influencing surgeons’ adherence to excisional margins recommended by the international guidelines—Univariate analysis

1% of 1583 interventions (86 missing values) respected the size of excisional margins recommended by guidelines on both Deltas (data not showed). Delta minor reflected the recommended guidelines only in 10% (153) of surgical procedures on the excisional margins, while the Delta major was in line with the guidelines in 54% (901) of surgical procedures.

Table 2 shows the AEMR for Delta major and Delta minor.

**Table 2:** Correlation between the adherence to the guidelines versus clinical variables and demographic data of patients.

Variables		AEMR… ^a^					p^b^
		Delta major		p^c^	Delta minor		
		No N(%)	Yes N(%)		No N(%)	Yes N(%)	
Total		756(46)	901(54)		1434(90)	153(10)	
Diagnosis	BCC	371(31)	831 (69)	**<0.001**	1096(95)	56 (5)	**<0.001**
	SCC	388 (85)	70 (15)		338(78)	97(22)	
Anatomical localization of the lesions	I	251 (48)	271(52)	**<0.001**	444(88)	58(12)	0.182
	B	230 (38)	380(62)		521(91)	53(9)	
	H	278 (53)	250(47)		469(92)	42(8)	
Surgical reconstruction techniques	flap A	82(57)	61(43)	**<0.001**	121(87)	18(13)	**<0.001**
	flap R	76(55)	63(45)		124(92)	11(8)	
	flap T	29(57)	22(43)		38(79)	10(21)	
	graft	149(72)	58(28)		147(74)	53(27)	
	primary closure	423(38)	697(62)		1004(94)	61(6)	
Patient gender	M	485(47)	546(53)	0.167	897(90)	96(10)	0.963
	F	274(44)	355(56)		537(90)	57(10)	
Patient age (years)	≤75	289(36)	516(64)	**<0.001**	723(95)	42(6)	**<0.001**
	76–85	269(50)	272(50)		452(88)	64(12)	
	≥86	201(64)	113(36)		259(85)	47(15)	
Surgeon experience (years)	<10	276(39)	425(61)	**<0.001**	617(93)	47(7)	**0.003**
	≥10	483(50)	476(50)		817(89)	106(12)	
Patients with margin interested	no	704(45)	876(55)	<0.001	1370(91)	140(9)	0.027
	yes	55(69)	25(31)		64(83)	13(17)	
Patients with margin interested ^**c**^ (N = 81)	Lateral	25(71)	10(29)	0.576	30(86)	5(14)	^e^
	Deep	20(63)	12(38)		23(79)	6(21)	
	Both	10(77)	3(23)		11(85)	2(15)	

^a^ AEMR (adherence to excisional margins recommended by the international guidelines) reports if the Delta (Delta is an estimate of the measure of excisional margins observed) reflects the guidelines or not. The guidelines defined the size that the standard excisional margins must to be in order to guarantee a complete removal of tumor in 95% of cases (see Methods).

^b^ p-value of χ^2^ test: the null hypothesis was that the groups considered in the “Variables” column have the same percentage of cases.

bold: p-value <0.05.

^c^ These variables was classified only on patients with margins interested: N = 81.

^e^ the chi-square test is not computable: 33.3% have expected count less than 5 and the minimum expected count is 2.19.

The percentage of adherence to excisional margins as recommended by the guidelines for Delta major was significantly higher in: BCC tumors (N = 831, 69%), B region tumors (N = 380, 62%), in primary closure (N = 697, 62%) and flap R reconstruction techniques (N = 63, 45%), in younger patients (≤ 75 years) (N = 516, 64%), and in less experienced surgeons (< 10 years) (N = 425, 61%).

The percentage of adherence to excisional margins as recommended by guidelines for Delta minor was significantly higher in: SCCs tumors (N = 97, 22%) compared to BCC tumors group (N = 56, 5%), in grafting and flap T reconstruction techniques (N = 53, 27% in grafting group; N = 10, 21% in the flap T group), in older patients (patients aged > 76 years old) (N = 111, 27%), and experienced surgeons (N = 106, 12% experienced surgeons of ≥ 10years; N = 47, 7% for < 10 years).

Out of 1669 excisions, 81 (5%) showed incomplete excision, thus being not radical. Among these, 35 of the involved margins were radial (43%), 33 deep (41%), and 13 both radial and deep (16%). However, the univariate analysis was not computable for these three groups because the assumptions of Chi-square test were not satisfied. Eighty-one patients with involved margins underwent re-excision according to the clinical procedure of the Department.

### Factors influencing surgeons’ adherence to excisional margins recommended by the international guidelines and completeness of excision—Multivariate analysis

Table 3 shows the first logistic regression model. The outcome of the model is “Adherence to excisional margins recommended by the international guidelines” referred to Delta minor.

**Table 3:** Logistic regression model of dependent variable “follow the adherence to excisional margins recommended by the international guidelines” concerning the Delta minor.

Covariates		AEMR Delta Minor^a^		
		OR	CI95% (OR)	
			inf	sup
Diagnosis	SCC	1		
	BCC	**0.22**	**0.15**	**0.31**
Anatomical localization of the lesion	H	0.60	0.40	0.90
	B	**1.77**	**1.13**	**2.90**
	I	1		
Patient gender	M	1		
	F	1.10	0.76	1.59
Patient age (years)	≤75	1		
	76–85	**1.72**	**1.12**	**2.65**
	≥86	**1.61**	**1.000**	**2.59**
Surgeon experience (years)	<10	**1**		
	≥10	**1.98**	**1.32**	**2.98**
Involved margins	No	1		
	Yes	1.48	0.75	2.90
Hosmer and Lemeshow’s test				0.398

^a^ It is the outcome of the model: it reports if the Delta minor (Delta minor is an estimate of the measure of excisional margins observed) reflects the guidelines or not. (AEMR for Delta minor).

Bold: p<0.05.

The factors that are significantly associated with recommended excisional margins for Delta minor are as follows: excisions of SCCs rather than BCCs (OR = 0.22; CI95%: 0.15–0.31), treatments of tumors localized in the B area (OR = 1.77; CI95%: 1.13–2.90), older patients (76–85 years old OR = 1.72 CI 95%: 1.12–2.65 and ≥86 years old OR = 1.61 CI95%: 1.00–2.59), and surgeons with more experience (experience ≥10 years: OR = 1.88; CI95%: 1.25–2.83).

The goodness of fit for this model was p = 0.398 of the Hosmer and Lemeshow’s test.

The second logistic model considered the completeness of excision (at deep margin, lateral margin or both) as outcome (see Table 4).

**Table 4:** Logistic regression model of dependent variable “incomplete excision”.

Covariates		Incomplete excision (lateral, deep or both)		
		OR	CI95% (OR)	
			inf	sup
Diagnosis	BCC	0.73	0.43	1.23
	SCC	1		
Anatomical localization of the lesion	H	1.39	0.83	2.32
	B	**0.31**	**0.16**	**0.60**
	I	1		
Patient gender	M	1		
	F	1.02	0.63	1.65
Patient age (years)	≤75	1		
	76–85	0.69	0.41	1.16
	≥86	1.10	0.60	1.99
Surgeon experience (years)	<10	1		
	≥10	1.03	0.60	1.75
Delta major according to guidelines ^a^	no	1		
	yes	**0.33**	**0.20**	**0.56**
Delta minor according to guidelines ^a^	no	1		
	yes	1.22	0.62	2.39
Hosmer and Lemeshow’s test				0.457

^**a**^ The Delta is an estimate of the measure of excisional margins: the variable reports if the measure is in conformity with the guidelines.

Bold: p<0.05.

As indicated in Table 4, it is more likely to find incomplete excisions in tumors localized in the H area (OR = 1) and in SCCs (OR = 1.44; CI 95%: 0.85–2.45).

The p-value of the Hosmer and Lemeshow’s test referred to the model is p = 0.457.

## Discussion

Understanding the factors that may contribute to poor standards of care is most important. There are no formal data to document and to explain why treatment guidelines are not followed. It has been hypothesized that reasons for poor adherence to guidelines in daily practice may be considered such as strength of habit without any identified reason, lack of knowledge or confidence in treatment guidelines and concerns about safety in fragile patients [14].

In the present research, the univariate analysis shows that the mean value of both Deltas (measured in mm) is associated to some demographic characteristics of patients such as gender and age. Mean values are significantly lower in female and in younger patients.

Hajjaj et al [15] reviewed the influence of non-clinical factors on medical decisions. Although most clinical decisions are based on ‘traditional’ clinical criteria, they are also influenced by a wide range of non-clinical factors, such as patients’ socioeconomic status, race, age, gender, and other personal characteristics. Gender can play an apparently inappropriate role in clinical decision-making. For example, women receive more laboratory tests, blood pressure checks, drug prescriptions, physical examinations and return appointments than men. Women also have more physician visits per year and more services per visit [16]. The findings of the present analysis show that Deltas are reduced in women, suggesting that surgeons might be influenced by gender when deciding the choice of surgical approach. Surgeons probably take into account the need for the scar to be as small as possible when operating on women motivated by aesthetic outcomes and cultural reasons [17].

Age can influence the clinical management [15]. Some studies show that the coexisting medical conditions in older people can adversely affect surgical care and surgical outcomes. Moreover, old age itself is an important risk factor for surgical candidates [18].

The findings reported show the opposite: the size of both Deltas increased proportionally with age. In this study, older people received more appropriate treatment. The possible explanation is that the increase in Deltas may ensure excisional margins free from neoplastic cells and result in a desirable decrease in additional re-excision procedures in elderly frail patients.

The mean value of both Deltas is also associated to the anatomical localization of NMSC. The data shows that tumors in the I and B areas had the higher mean value for Deltas compared to lesions localized in the H area. It is well documented that H area tumors have a higher recurrence and metastasis rate [19]. Despite such an unfavorable clinical feature, surgeons are reluctant to respect the standard excision margin in this area. Previous studies indicate the presence of incomplete excisions in the same anatomical complex areas for SCC tumors [20,21].

The reason might be that surgeons tend to save tissues in order to avoid larger scars on cosmetically important units, or to avoid creating functional damage in perioral or periorbital regions usually requiring complex reconstructions. Similar results, published in 2009 by the British Association of Aesthetic Plastic Surgeons, showed that the scalp had a slightly higher excision margin compared to the more cosmetically sensitive parts of the face [20]. Moreover, scar revisions are one of the most requested aesthetic procedures. Therefore, particularly on the face, surgeons tend to leave a scar that will not require any surgical revision [22].

There are, however, differences between Delta major and Delta minor associated to reconstruction technique, histologic type of cancer, and surgeons’ experience.

Elliptical excisions showed a higher Delta major compared to the other ones (p<0.001); on the contrary, the grafting technique had a higher Delta minor (p<0.001).

We hypothesize that the reason for such results may be related to the surgical design. The ellipse is drawn so that the length of the ellipse is at least three times the width of the ellipse, to facilitate the optimal closure of the wound and to prevent formation of the cutaneous cones [23]. The surgical design aims at maintaining the ratio between major axis and minor axis. The latter is designed to correspond to the Delta minor. In fact, the average size of our Delta major is 11.0 mm, well above the standard excisional margin, but equivalent to approximately three times the average Delta minor.

However, the Delta minor is higher in case of defect reconstructed with skin grafting. Skin grafting is not affected by the limits of the availability of donor area such as flaps or primary closure and allows the excision of wider surgical margins [24].

Ultimately, SCCs are the tumors with the higher Delta minor. Compared to BCCs, SCCs tend to be more invasive and/or metastatize more frequently. Although a causal nexus cannot be established in this study, a possible explanation is that the surgeon usually chooses a relatively broader margin in SCCs in order to avoid local recurrence and metastasis.

This data is further confirmed by the result concerning the risk class where the minor Delta is significantly higher in high risk BCCs and SCCs compared to low risk BCCs and SCCs (p<0.001). It is right to point out that in the case of high-risk SCCs the minor Delta respected the size of excisional margins recommended by guidelines (6–10 mm in the case of SCCs R+).

Regarding the correlation between Deltas and surgeons’ experience, a higher Delta minor is found with experienced surgeons (≥ 10 years) (p<0.001). Experience leads to compliance with recommendations on margins despite the surgical type of reconstruction and the location. This trend of better outcomes is noticeable for many surgeons in different types of surgery [25,26].

Univariate analysis was employed to investigate whether the same clinical and non-clinical factors may affect compliance with excisional margins recommended by the international guidelines.

As shown in Table 2, Delta major usually corresponds to the international guidelines, whereas with Delta minor there is a lower degree of compliance.

The results of this study show that the surgical procedures where adherence to the standard Delta minor is achieved are as follows: 1) older patients (≥ 75 years); 2) grafting and flap T reconstruction techniques; 3) SCC tumors; 4) experienced surgeons.

The logistic analysis in Table 3 evaluates the conformity of Delta minor to recommended excisional margins, taking into account the simultaneous effect of all the co-variates. Interestingly, with this kind of analysis we see similar results as in the univariate analysis. In particular, the recommended excisional margins for Delta minor are implemented to a greater extent when: patients are older, dealing with SCCs tumors, surgical procedure is carried out by an experienced surgeon, the tumor is located in the B area. Based on the reported results, the same factors affecting the Delta minor mean value affect the compliance with recommended excisional margins both with univariate and multivariate analysis, confirming the association reported in Table 1.

A second logistic model (Table 4) evaluates completeness of excisions. In this study the percentage of incomplete excisions is very low (5%) despite the percentage of non-compliance with the guidelines. However, the data matches the average rates of incomplete excision showed in previous studies [27].

SCCs have the highest probability to be incompletely excised despite the fact that excisions of SCCs tend to reflect the recommended excisional margins for Delta Minor to a greater extent than BCCs. This can be due to the fact that SCCs usually develop on sun-exposed skin and can be associated to actinic keratosis, resulting in the so-called cancerization field. This clinical presentation can pose difficulties in identifying the tumor margins [28].

Similarly, the localization of the tumor plays an important role in the presence of incomplete excision: excisions performed in the B area are more likely to reflect the recommended excisional margins and complete excisions are usually found in this area. The H region shows a more complex anatomy causing a higher rate of incomplete excisions, probably due to the attempt to preserve underlying vital anatomical structures (cartilage, facial nerves).

Similarly to these results, other studies also indicate the presence of incomplete excisions in the same anatomically complex areas for SCC tumors [20,21].

### Strengths and limitations

A standardized approach allows the study to be replicated in different areas or over time with the production of comparable findings.

Findings are subject to several limitations. Biases of this retrospective study might be the fact that comorbidity has not been evaluated along with skin photo-type. Also tumor size and histologic specimen size were only evaluated ex vivo by pathologists. Although there is no standardized value for the shrinkage phenomenon, a mean shrinkage of 17% in the length and 9.5% in the width was used [13]. However, some biases may have occurred.

### Conclusion

Our data shows that only 1% of cases achieved an overall compliance with the international guidelines. Although this percentage is not high, numerical data shows that also in cases where the Delta was not sufficient, its average value was close to the recommended value. Moreover must be underlined that adherence to guide lines was appropriate in high risk SCC. This suggests that surgeons respect the recommended excisional margins and in the more risky cases and in general attempted to comply with the existing guidelines.

It was shown that both demographics and clinical factors influence surgeons’ adherence to the guidelines for excisional margins.

The results show that (1) a histological diagnosis of SCC, (2) an experienced surgeon, and (3) old age are the determining factors that impact positively on the adherence to the recommended guidelines. Under the same conditions of analysis, tumor localization in the H area has the strongest negative impact.

The identification of factors influencing the adherence to the recommended guidelines sheds light on clinicians’ practice and decision-making regarding excisional margins. Hopefully this data will promote correct surgical behavior in the future in order to achieve greater compliance with the guidelines.

## Supporting information

S1 File Dataset-Supporting information files07-08-2018.sav. (SAV)
